# Muramyl dipeptide CD10 monoclonal antibody immunoconjugates inhibited acute leukemia in nude mice

**DOI:** 10.1042/BSR20222668

**Published:** 2023-04-25

**Authors:** Yilin Wang, Xiaofu Jin, Yan Sun, Yanxia Zhao, Zhenghai Qu, Lingzhen Wang, Lirong Sun

**Affiliations:** 1 Department of Pediatric Hematology, The Affiliated Hospital of Qingdao University, Shandong 266003, PR China; 2 Department of Pediatric Internal Medicine, Jinhua Municipal Central Hospital Medicine Group, Jinhua, Zhejiang 321000, PR China; 3 Department of Pediatric, The Affiliated Hospital of Qingdao University, Shandong 266003, PR China

**Keywords:** acute lymphoblastic leukemia, anti-CD10 mAb, immunotherapy, minimal residual disease, muramyl dipeptide

## Abstract

Minimal residual disease (MRD) is one of the causes of leukemia recurrence. Previously, we developed anti-CD10 mAb conjugated to muramyl dipeptide immunoconjugate (MDP-Ab) for immune enhancement. The present study aimed to investigate anti-leukemia effect of MDP-Ab administered via different methods in leukemia ectopic graft nude mouse model. BALB/c nude mice were injected with Nalm-6 cells subcutaneously to establish leukemia xenografts in nude mice as a model. MDP-Ab or/and human lymphocytes (LYM) was injected into different sites of the nude mice. Immunohistochemistry staining of CDs in the bone marrow, liver and spleen was performed. IFN-γ was detected by ELISA. We detected the metastasis of leukemia cells to the liver, spleen and bone marrow in nude mouse leukemia model. MDP-Ab and LYM inhibited the growth of tumors, and simultaneous injection of MDP-Ab and LYM into the tumor inhibited the growth of tumors. IFN-γ levels in MDP-Ab (ca) + h-LYM (ca) group, MDP-Ab (ca) + h-LYM (ip) group, MDP-Ab (iv) + h-LYM (ip) group and PBS (ca) + h-LYM (ca) group were significantly higher than those in control group, while IFN-γ level in MDP-Ab (ca) + h-LYM (ca) group was the highest. Moreover, MDP-Ab and h-LYM promoted the expression of hCD4 and hCD8, with the highest expression in MDP-Ab (ca) + h-LYM (ca) group. In conclusion, MDP-Ab effectively promoted the production of IFN-γ, enhanced the antitumor immunity of T lymphocytes and inhibited leukemia.

## Introduction

Acute lymphoblastic leukemia (ALL) is the most common type of leukemia, and approximately 80% of ALL cases are acute B-lymphoblastic leukemia (B-ALL) [1–3]. With the continuous development of chemotherapeutic drugs and improvement of chemotherapeutic regimens, the overall survival rate of children with ALL has reached 80–90% [4,5]. However, 15–20% of children still relapse after the first remission. Studies have shown that minimal residual disease (MRD) is the main cause of recurrence [6]. Therefore, MRD is an important prognostic indicator. MRD refers to the presence of a small number of leukemia cells that cannot be detected by conventional cytomorphological methods in leukemia patients after induced remission or bone marrow transplantation [1,7]. To improve the prognosis and survival rate of children with ALL, a treatment strategy of effectively eliminating MRD leukemia cells and preventing recurrence must be adopted. Therefore, the successful establishment of an MRD animal model is important. Nude mice (BALB/c-nu mice) have abnormal T lymphocyte maturation due to loss of the thymus, resulting in abnormal T lymphocyte function, and they are often used to establish blood disease models.

Bacillus Calmette Guerin (BCG) is a live attenuated Mycobacterium tuberculosis vaccine that is considered to be one of the safest vaccines for preventing Mycobacterium tuberculosis infection [8]. BCG is not only a safe vaccine but also an immune adjuvant that promotes the secretion of cytokines and enhances the function of immune cells *in vivo*. To date, studies have reported that BCG can be used in the treatment of bladder cancer [8–10]. However, there have been few studies on the application of BCG in the treatment of blood tumors. Our group identified that muramyl dipeptide immunoconjugate (MDP), an immunogenic molecule from BCG, has the characteristics of low toxicity and high activity and is the smallest effective molecule with immune activity in *Mycobacterium tuberculosis* [11].

CD10 is a neutral endopeptidase with enzymatic activity that can hydrolyze the peptide chain to stimulate B lymphocyte differentiation and is highly expressed in most pre-B-ALL cells. Studies have shown that the expression of CD10 is related to MRD and indicates a favorable prognosis, so it is used for the detection of MRD [12,13]. By using MDP from BCG as an antigen, our group combined the antigen with an antibody against CD10 expressed on the surface of B-ALL leukemia cells so that the combination could be recognized by cytotoxic T lymphocytes (CTLs) after entering the body. As CD10 antibody binds to CD10 on the surface of leukemia cells *in vivo*, CTLs can be activated to kill leukemia cells. At the same time, leukemia cells can be eliminated through complement-dependent cytotoxicity (CDC) and antibody-dependent cellular cytotoxicity (ADCC). Previously, we reported that an anti-CD10 mAb conjugated to MDP (MDP-Ab) exhibited targeted characteristics and immune enhancement [13–15]. However, the *in vivo* activity of MDP-Ab remains unclear. Therefore, the present study aimed to establish a nude mouse model of MRD leukemia and explore the antileukemia effects of MDP-Ab *in vivo*.

## Materials and methods

### Cell culture

Nalm-6 cells, a human acute pre-B lymphocyte leukemia cell line identified by STR (Sai Bai-kang, Shanghai, China), were cultured in RPMI 1640 (Gibco, Grand Island, NY, U.S.A.) supplemented with 10% (v/v) fetal bovine serum (BI) and 1% (v/v) penicillin (BI) in a humidified incubator with 5% CO_2_ at 37°C, and exponentially growing cells were used for the experiments.

### Preparation of human peripheral blood lymphocytes

Lymphocytes were extracted from six healthy volunteers inoculated with the BCG vaccine or patients with tuberculosis. Total 5 ml of blood samples were taken and 5 × 10^6^ lymphocytes were isolated from each volunteer. Written informed consent was obtained from the participants according to the Declaration of Helsinki, and the protocols were approved by the Ethics Committee of the affiliated hospital of Qingdao University. Lymphocytes were separated by density gradient centrifugation with human lymphocyte separation solution (Solarbio, Beijing, China). The cells were resuspended in RPMI-1640 at a density of 1 × 10^7^/ml for subsequent experiments.

### Preparation of MDP-Ab

The MDP-Ab was synthesized as described previously [15]. The concentration was adjusted to 20 µg/0.1 ml for intratumoral injection and caudal vein injection. The volume of all injections at each site was 0.1 ml.

### Establishment of the leukemia model

Female BALB/c-nu mice (4–5 weeks old, weight 12–15 g) were purchased from Jinan Pengyue Experimental Animal Breeding Co., Ltd. (license number: SCXK (lu)20190003). All animal experiments took place at the affiliated hospital of Qingdao University and housed in a specific pathogen-free laboratory. And all animal procedures were performed in accordance with the ARVO Statement for the Use of Animals, and approved by Ethics Committee of the affiliated hospital of Qingdao University (approval number: QDFY-2020-01-03). An *in vivo* ectopic tumor model was established with subcutaneous injection of 2 × 10^6^ nalm-6 cells into the right axilla of female BALB/c-nu mice. The formation and growth of solid tumors (mm) were measured with metric calipers at 2- to 3-day intervals using the following formula: *V* = (*L* × *S*2)/2, where *L* represents the longer tumor diameter and *S* represents the smaller tumor diameter. BALB/c-nu mice were randomly divided into five groups (6 mice in each group) after the tumor volume reached approximately 56 ± 10 mm^3^ as follows: MDP-Ab(iv)+h-LYM(ip) (BALB/c-nu mice were injected with MDP-Ab intravenously and human lymphocytes intraperitoneally), MDP-Ab(ca)+h-LYM(ip) (BALB/c-nu mice were injected with MDP-Ab into the tumor, and human lymphocytes were injected intraperitoneally), MDP-Ab(ca)+h-LYM(ca) (MDP-Ab and human lymphocytes were both injected into the tumor), PBS(ca)+h-LYM(ca) (BALB/c-nu mice were injected with PBS and human lymphocytes intratumorally), and control group (only PBS was injected into the tumor). Each mouse was injected with lymphocytes from one of six volunteers. The volume of all injected substances was 0.1 ml and each mouse was only injected once during the whole experiment.

### Growth curve of tumors

The activity, diet, weight changes, mental state, urine and stool of leukemia nude mice were observed. Survival of the nude mice and tumor volume were monitored regularly, and a growth curve of solid tumors was generated.

### Enzyme-linked immunosorbent assay (ELISA)

Blood samples were collected after anesthesia. Serum levels of interferon γ (IFN-γ) of nude mice were measured with ELISA kit (Jiangsu Jingmei Biotechnology Co., Ltd) according to the manufacturer’s instructions. The absorbance of each well was measured at a wavelength of 450 mm. All experiments were repeated three times.

### Hematoxylin and eosin (HE) staining

The nude mice with leukemia were killed at the 15th day after cell immunization, by cervical dislocation after inhalation of isoflurane anesthesia, and the tumors were fixed in 4% paraformaldehyde solution for at least 24 h and embedded in paraffin. Then, they were cut into 5 µm thick slices, dewaxed and hydrated. The sections were stained with hematoxylin–eosin and washed with PBS five times. Images were obtained with a microscope.

### Immunohistochemistry

Paraffin sections were dewaxed and hydrated, and antigen repair was performed with 0.01 mol/L citrate buffer. Paraffin sections were blocked with endogenous peroxidase blockers. Nonspecific antigen was blocked with 10% calf serum. Subsequently, paraffin sections were incubated overnight at 4°C with the rabbit antibodies against human CD4 (Abcam, ab133616, Cambridge, U.K., 1:250), human CD8 (Abcam, ab4055, Cambridge, U.K., 1:200), human CD19 (Abcam, ab134114, Cambridge, U.K., 1:20), mouse CD14 (Abcam, ab182032, Cambridge, U.K., 1:1000) and mouse CD56 (Abcam, ab220360, Cambridge, U.K., 1:2000). The following day, HRP-labeled goat anti-rabbit secondary antibodies (Abcam Cambridge, U.K.) were incubated with the tissues at room temperature for 1 h. After staining with DAB chromogenic solution (Beijing Zhongshan Jinqiao Biotechnology Co., Ltd., China) at room temperature, the sections were observed under a light microscope. All experiments were repeated three times.

### Statistical analysis

Statistical analysis was performed using IBM SPSS V 25 and GraphPad Prism 7. All data are expressed as the mean ± standard deviation. Differences among multiple groups were analyzed using one-way ANOVA, and a value of *P*<0.05 was considered statistically significant.

## Results

### Establishment of leukemia mouse model

**Figure 1:**
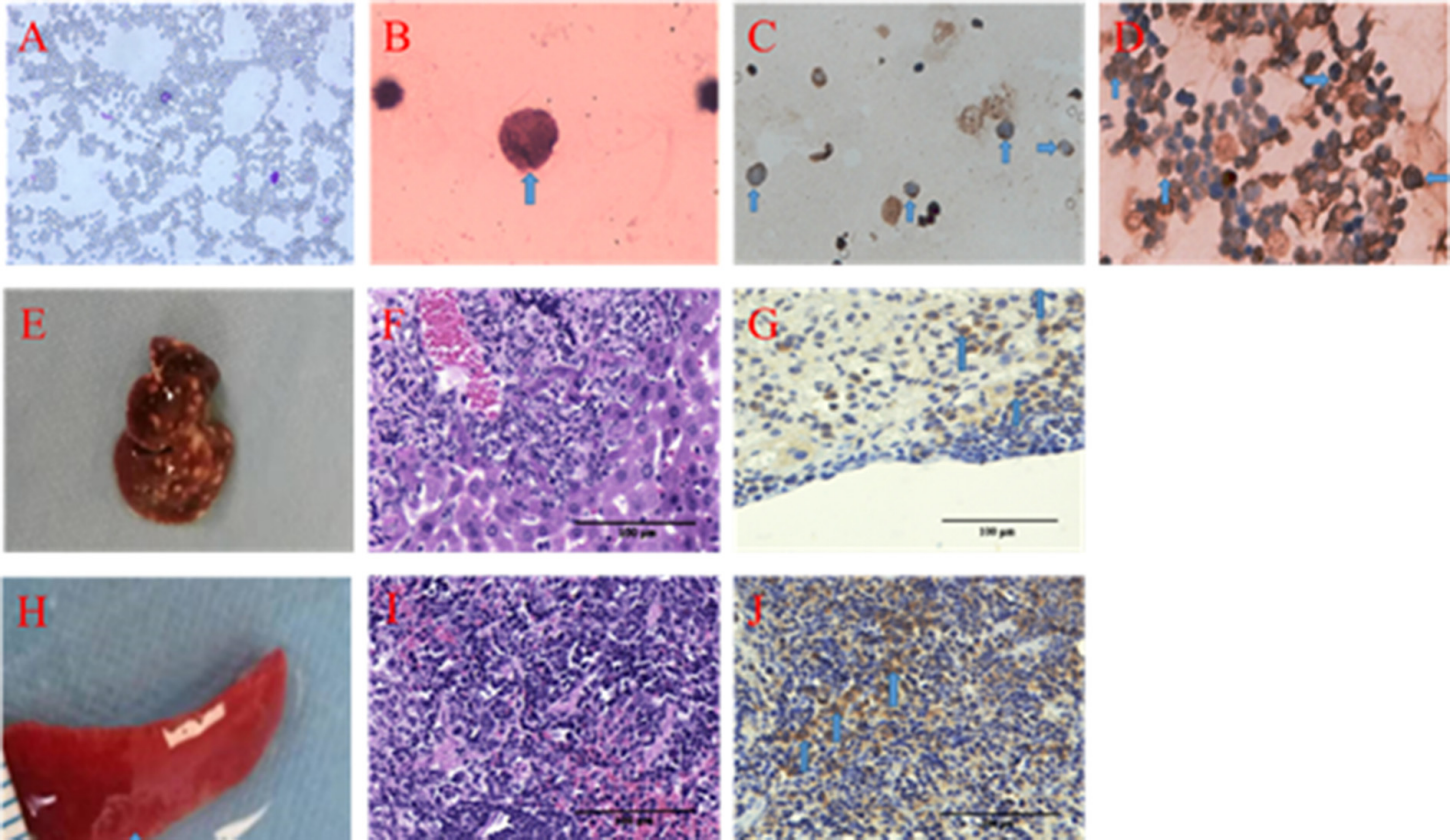
The leukemia xenografts model in nude mice (**A**) HE staining of peripheral blood (400×). (**B**) HE staining of bone marrow (1000×). (**C**) Positive staining of CD19 in bone marrow (400×). (**D**) Positive staining of CD10 in bone marrow (400×). (**E**) A white mass structure was observed on the surface of the liver. (**F**) HE staining of the liver. (**G**) Positive staining of CD19 in the liver. (**H**) White ring structure on the surface of the spleen. (**I**) HE staining of the spleen. (**J**) Positive staining of CD10 in the spleen.

First, we successfully established leukemia xenografts in nude mice as a model. At 11.0 ± 2.5 days after subcutaneous injection, a tumor with a size of 3–4 mm was observed at the injection site. HE staining demonstrated that large cellular abnormal lymphocytes with large nuclei and light staining were observed in the bone marrow There were no abnormal lymphocytes in peripheral blood, but a large number of red blood cells and a few neutrophils had lobulated nuclei (Figure 1A,B). CD19 and CD10 immunohistochemistry were performed on the bone marrow (Figure 1C,D), and the cells were CD19 (+) and CD10 (+), which further suggested that the heterotypic cells were human Pre-B-ALL leukemia cells.

In addition, we dissected the liver and spleen of nude mice. A white mass structure was found on the surface of the liver (Figure 1E). HE staining indicated that the normal structure of the liver was destroyed, and there were clumps of abnormal cells (Figure 1F), which were immunohistochemically CD19 (+) (Figure 1G). We also found abnormal white ring structures on the spleen (Figure 1H), and abnormal cells with large and shallow nuclei were shown by HE staining (Figure 1I). Immunohistochemistry showed CD10 positivity (Figure 1J), suggesting metastatic leukemia cells in both the liver and the spleen.

### MDP-Ab exerted antileukemia effects *in vivo*

**Figure 2:**
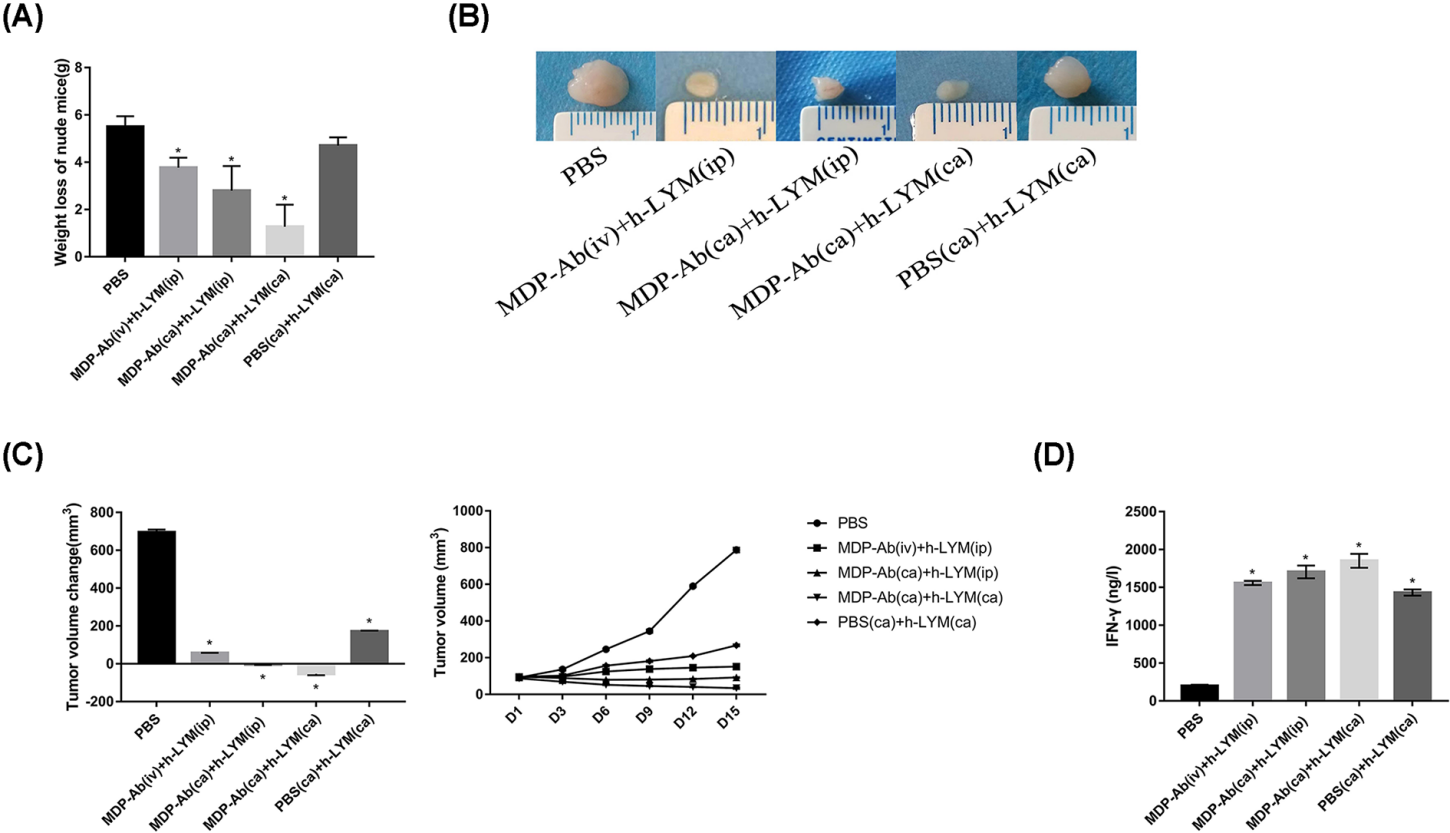
MDP-Ab inhibited the growth of tumors in nude mice (**A**) Compared with the first day, the weight changes of nude mice in different groups on the 15th day (*n*=6). (**B**) The subcutaneous xenograft tumors in different group on the 15th day. (**C**) The volume changes of tumors in different groups of nude mice and the changes on the 15th day compared with the first day (*n*=6). (**D**) Detection of IFN-γ levels in different groups of nude mice by ELISA. *P<0.05 vs. PBS (*n*=6).

We found no significant differences in the activities and diet of nude mice before and after modeling, and there was no hematuria or bloody stool. Compared with the first day, on the 15th day the weight loss of the control group was the greatest (5.50 ± 0.44 g), followed by the PBS (CA) + h-LYM (ca) group, MDP-Ab (iv) + h-LYM (ip) group and MDP-Ab (ca) + h-LYM (ip) group. The weight loss of the MDP-Ab (ca) + h-LYM (ca) group was the lowest, which was 1.27 ± 0.93 g (Figure 2A). Subsequently, we measured the volume of tumors after different injection methods. As expected, MDP-Ab inhibited tumor growth, and the tumor volume decreased most significantly in the MDP-Ab(ca)+h-LYM (ca) group, with a reduction of 53.04 + 7.20 mm^3^ on the 15th day compared with the first day. However, MDP-Ab intravenous administration and intraperitoneal injection of human lymphocytes did not effectively prevent tumor proliferation (Figure 2B,C).

We also detected IFN-γ level in nude mice by ELISA. The results showed that both MDP-Ab and human lymphocytes increased the level of IFN-γ. The level of IFN-γ in the MDP-Ab (ca) + h-LYM (ca) group was (1850.5 ± 91.91) ng/l, significantly higher than the (199.85 ± 13.28) ng/l in the control group, (1704.23 ± 84.7) ng/l in the MDP-Ab(ca)+h-LYM(ip) group, (1558.04 ± 28.22) ng/l in the MDP-Ab(iv)+h-LYM(ip) group and (1430.61 ± 40.68) ng/l in the PBS(ca)+h-LYM(ca) group (Figure 2D).

### MDP-Ab and h-LYM promoted T-cell activation

**Figure 3:**
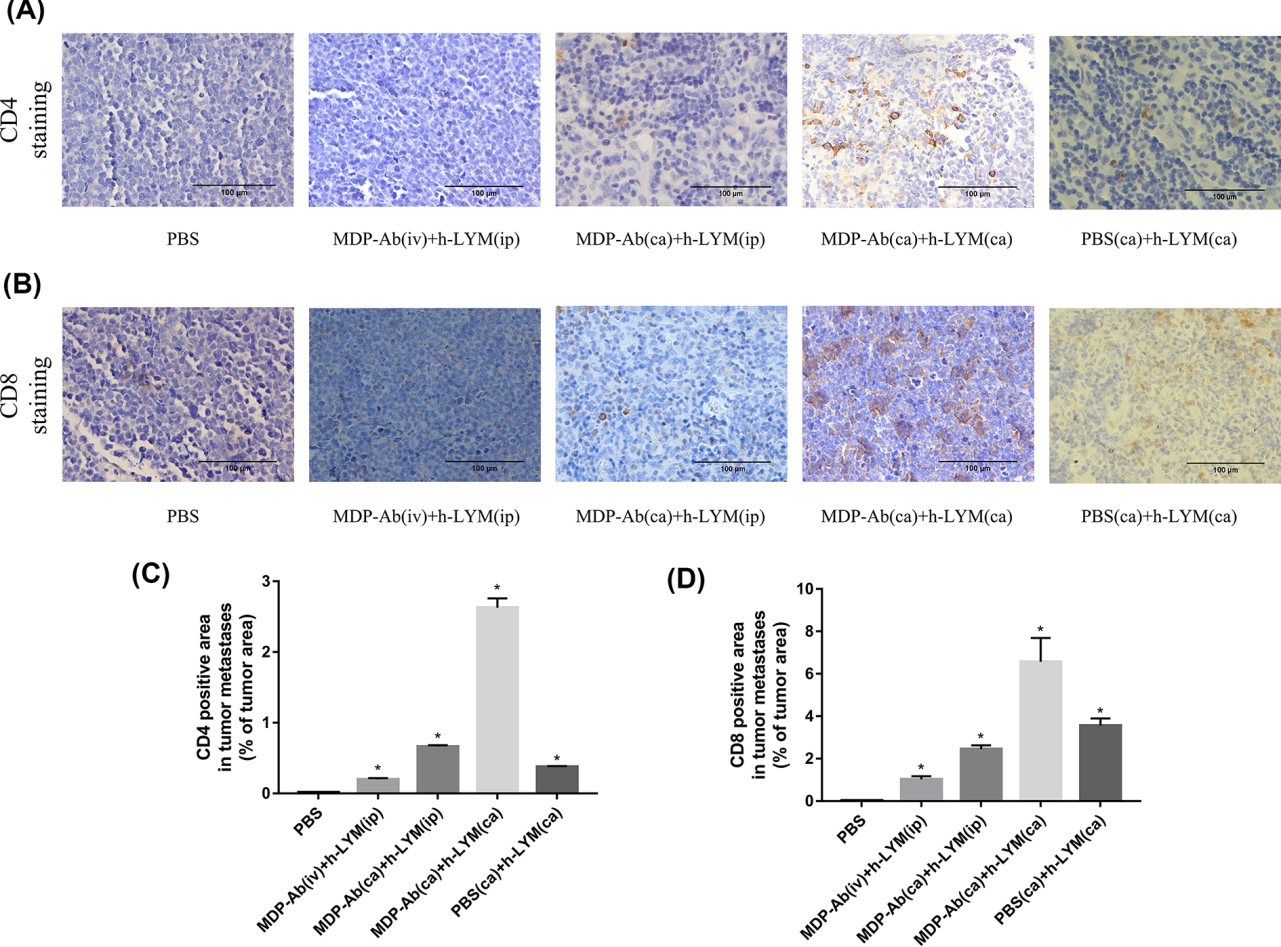
The level of CD4- and CD8-positive T-cell infiltration in the tumor 15 days post-injection Immunohistochemistry staining of CD4 (**A**) and CD8 (**B**) T-cell regions in nude mice from different groups with tumors. The results were shown as the percentage of tumor area (%) (staining area/ tumor area), the percentage of CD4-positive cells (**C**) in different groups and the percentage of CD8-positive cells (**D**) in different groups. *P<0.05 vs. PBS (*n*=6).

Next, we explored the potential mechanism by which MDP-Ab inhibits leukemia. We detected the expression of T lymphocyte surface antigens by immunohistochemistry and observed that human CD4 (hCD4) and human CD8 (hCD8) were activated by MDP-Ab and human lymphocytes. We validated the expression of CD4 and CD8 in different groups and found that the expression of CD4 and CD8 increased most significantly during intratumoral injection of MDP-Ab and h-LYM (Figure 3A–D).

### Effects of MDP-Ab and h-LYM on other immune cells

**Figure 4:**
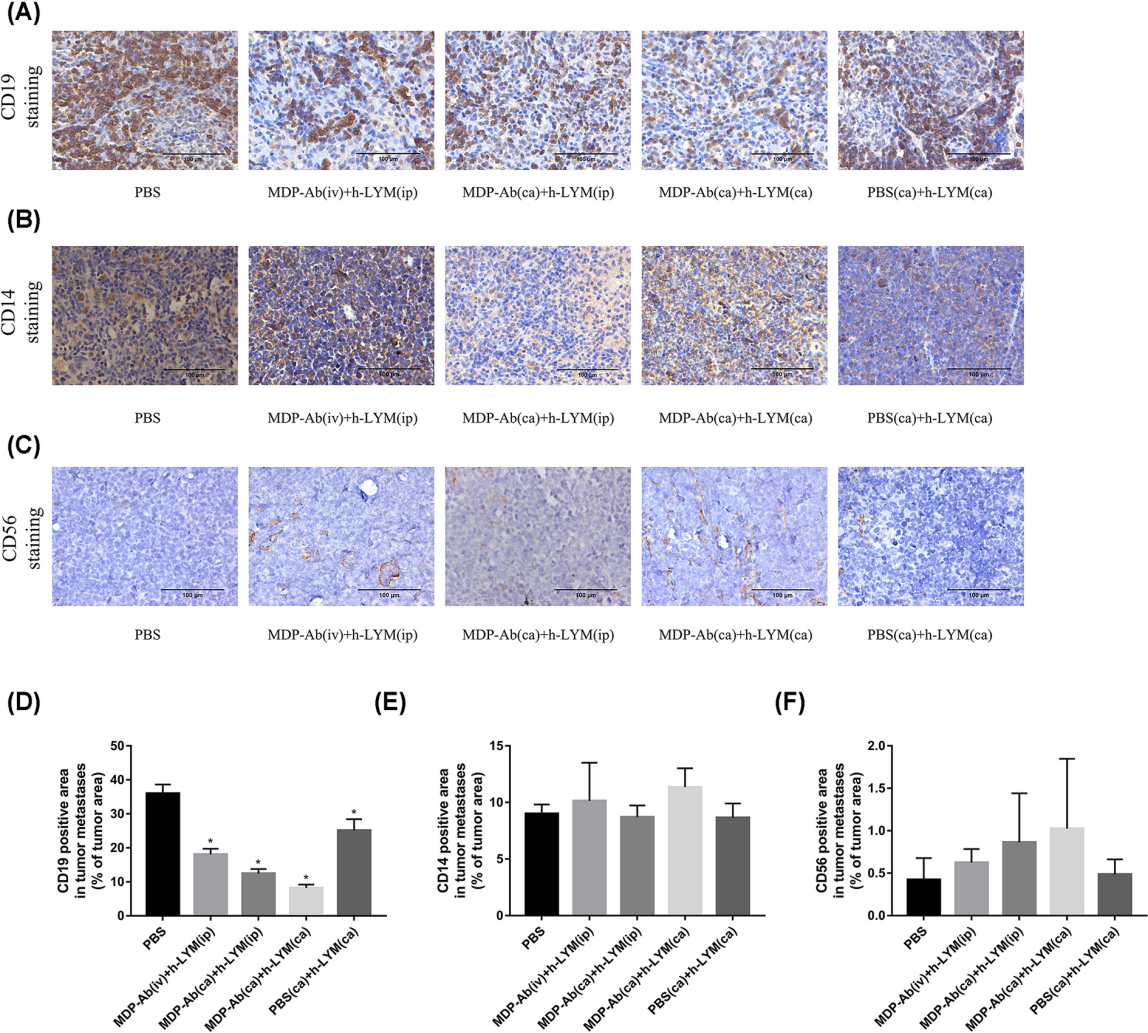
The infiltration levels of CD19-, CD14- and CD56-positive cells in tumors Immunohistochemistry staining of CD19 (**A**), CD14 (**B**) and CD56 (**C**). Positive cell regions were detected in nude mice from different tumor groups. The infiltration levels of CD19- (**D**), CD14- (**E**) and CD56-positive cells (**F**) in different groups were expressed as the percentage of tumor area (%) (staining area/tumor area). *P<0.05 vs. PBS (*n*=6).

Immunohistochemistry results revealed that MDP-Ab and human lymphocytes inhibited the expression of human CD19 (hCD19). The expression of CD19 increased in the following order: the MDP-Ab (ca) + h-LYM (ca) group, MDP-Ab (ca) + h-LYM (ip) group, MDP-Ab (iv) + h-LYM (ip) group, PBS (ca) + h-LYM (ca) group and PBS (ca) group (Figure 4A,D). We also detected the expression of mouse CD14 and mouse CD56 in the tumors and found no significant differences among the groups (Figure 4B,C,E,F).

## Discussion

In recent decades, the cure rate of children with B-ALL has steadily improved, but the treatment of relapsed/refractory B-ALL remains a challenge [16]. MRD is the main cause of relapse, and traditional cytotoxic chemotherapy is unlikely to improve MRD. Instead, emerging molecular and immunological treatments, including adoptive cellular immunotherapy, tumor vaccines and antibody therapy, show promise to treat MRD [17–19].

T-cell immunotherapy has become a hot topic in cancer immunotherapy in recent years [20–22], especially chimeric antigen receptor T-cell immunotherapy (CAR-T), which combines antigen-specific antibody properties and T-cell cytotoxicity. CAR-T cells utilize the capabilities of innate and adaptive immunity to specifically recognize extracellular tumor antigens [23–25]. The mechanism of action of MDP-antiCD10 conjugate is similar to that of CAR-T cells. However, since the scFv portion of the CAR-T complex is usually mouse derived, CAR-T cells have potential for immunogenicity. However, MDP-Ab does not have this problem, which provides a new strategy for the treatment of leukemia.

In previous studies, MDP-Ab-induced dendritic cells enhanced the killing activity of lymphocytes against leukemia cells in vitro and improved the antitumor immune response mediated by CTLs [13–15]. The antitumor mechanism of MDP-Ab is based on the conjugate: on the one hand, the MDP-Ab immunoconjugate binds to CD10 on the surface of Nalm-6 cells, specifically and efficiently targeting CD10+ cells; on the other hand, MDP-Ab immunoconjugates can be recognized by the intracellular pattern recognition receptor NOD2 (CARD15) to bind and activate memory T lymphocytes that induce T-cell proliferation and exert an antitumor effect.

In the present study, we subcutaneously inoculated Nalm-6 cells, and tumors were detectable after 11.0 ± 2.5 days. Bone marrow HE staining and immunohistochemical staining showed positive staining for hCD19 and hCD10, indicating that the xenogeneic tumor model carrying Nalm-6 leukemia cells was successfully established. In addition, HE staining of the liver and spleen showed that the normal structure of the liver and spleen was destroyed, and immunohistochemical staining showed hCD19(+) and hCD10(+). These results suggest that the subcutaneous leukemia model has distant metastasis. Memory T cells were able to enter autologous tumor grafts and peripheral nonlymphoid tumor tissue but not autologous normal skin grafts [26].

We induced immune-deficient nude mice to generate immune responses by injecting human lymphocytes to provide normal T lymphocytes and injecting MDP-Ab at the same time. The results showed that the active human lymphocytes and the MDP-anti-CD10 conjugate had an anti-leukemia effect. MDP-Ab immunoconjugate specifically targeted Nalm-6 cells, and then T cells recognized MDP-Ab and combined with it to activate memory T cells, which can induce T-cell proliferation, and then killed Nalm-6 cells to achieve antitumor effect. In addition, we used different injection sites and found that the simultaneous injection of MDP-Ab and human lymphocytes into the tumor achieved the most significant inhibition on tumor growth, and the tumor volume in the MDP-Ab (ca) + h-LYM (ca) group was significantly smaller than that in the PBS (ca) group (control group), MDP-Ab (ca) + h-LYM (ip) group, MDP-Ab (iv) + h-LYM (ip) group, and PBS (ca) + h-LYM (ca) group, which may be related to drug metabolism in vivo and a xenograft rejection immune response. Further experiments at a later stage are required to confirm the results.

IFN-γ is produced by activated CD8-positive T cells, NK cells and CD4-positive T cells and serves as an important immune regulator to protect against infection and tumors [27]. IFN-γ can enhance monocyte-macrophage antigen presentation and the killing activity of specific immune cells [28]. In clinical studies of leukemia, IFN-γ has a negative regulatory effect. The level of IFN-γ is low in primary leukemia, and IFN- γ level increased after leukemia remission [29]. In this study, we detected serum IFN-γ and found that MDP-Ab and human lymphocytes increased the level of IFN-γ. These data indicate that MDP-Ab inhibits leukemia by up-regulation of IFN-γ.

Effective immunotherapy depends on robust effector T-cell function, and CD4+ T cells mediate antitumor immunity primarily by assisting CD8+ cytotoxic T lymphocytes and antibody responses [30,31]. CD4 T cells can aid in the recruitment of CD8 T cells and the long-term maintenance of CD8+ memory T cells [32]. Based on IHC staining of xenografts, our results showed that the intratumoral injection of MDP-Ab and human lymphocytes enriched CD4+ T lymphocytes and CD8+ T lymphocytes in xenograft tumors to exert antitumor effects. Therefore, antileukemic effect of MDP-Ab may be related to the induction of CTL-mediated antitumor immune responses. In addition, MDP is known to induce the activation of NF-kB signaling, which plays an important role in antitumor immunity [33]. Therefore, MDP-Ab may be a reasonable choice for the clinical development of a new immunomodulatory agent for the treatment of leukemia.

In conclusion, MDP-Ab can induce T cells to differentiate into Th1 cells, secrete IFN-γ, and induce CTL-mediated antitumor immune response. MDP-Ab can be used as a therapeutic antitumor vaccine for the treatment of leukemia.

## Data Availability

All data are included in this manuscript.

## Abbreviations

ADCC: antibody-dependent cellular cytotoxicity
ALL: acute lymphoblastic leukemia
BCG: Bacillus Calmette Guerin
CDC: complement-dependent cytotoxicity
CTL: cytotoxic T lymphocyte
IFN-γ: interferon γ
MRD: minimal residual disease
